# Supplementary material for: Deletion of wfs1 Impairs Oligodendrocyte Precursor Cells Dorsal Distribution and Myelination Through the wfs1-hmgcs1 Axis in Zebrafish
Source: Biology (Basel). 2026 Aug 16;15(16):1402. doi: 10.3390/biology15161402 (PMC13510151; doi:10.3390/biology15161402)
Supplement: Supplementary file 1 [file biology-15-01402-s001.zip › File S1-Transcriptome sequencing data and differentially expressed genes (DEGs) in wfs1-- zebrafish at 5 dpf/2.KEGG/wfs1KOvsWT/ALL/src/dre00600.html]

dre00600


- dre:493602

- Up regulated genes

493602(1.70187950602732)

- dre:493602

- Up regulated genes

493602(1.70187950602732)

- dre:100330155

- Down regulated genes

100330155(-1.11687791561759)

- dre:100330155

- Down regulated genes

100330155(-1.11687791561759)

- dre:553981

- Down regulated genes

553981\_1(-1.0489627113989)

- dre:450083

- Up regulated genes

450083(2.11033262833863)

- dre:445250

- Down regulated genes

445250(-5.57498320723853) 445250\_1(-7.69337783807938)
- dre:100000684

- Down regulated genes

100000684\_1(-1.98120251794954)

- dre:493602

- Up regulated genes

493602(1.70187950602732)

Close
